# Diagnosis and Treatment of Neurocysticercosis

**DOI:** 10.1155/2009/180742

**Published:** 2009-08-27

**Authors:** Christina M. Coyle, Herbert B. Tanowitz

**Affiliations:** ^1^Department of Medicine, Division of Infectious Disease, Albert Einstein College of Medicine, Bronx, NY 10461, USA; ^2^Parasitology and Tropical Disease Clinic and Laboratory, Jacobi Medical Center, Bronx, NY 10461, USA; ^3^Department of Pathology, Division of Parasitology and Tropical Medicine, Albert Einstein College of Medicine, Bronx, NY 10461, USA

## Abstract

Neurocysticercosis, the infection caused by the larval form of the tapeworm *Taenia solium*, is the most common parasitic disease of the central nervous system and the most common cause of acquired epilepsy worldwide. This has primarily been a disease that remains endemic in low-socioeconomic countries, but because of increased migration neurocysticercosis is being diagnosed more frequently in high-income countries. During the past three decades improved diagnostics, imaging, and treatment have led to more accurate diagnosis and improved prognosis for patients. This article reviews the current literature on neurocysticercosis, including newer diagnostics and treatment developments.

## 1. Introduction

Neurocysticercosis (NCC) is a neurologic infection caused by the larval stage of the tapeworm *Taenia solium*. In the developing world, NCC, infection of the central nervous system (CNS) with the *T. solium* larvae, is the most common cause of acquired epilepsy [1–3]. Because of globalization, many clinicians in industrialized countries who are unfamiliar with NCC are now faced with managing this disease. Humans are the definitive hosts for this parasite, and swine are the intermediate hosts. The adult tapeworm develops in human hosts after they ingest live cysticercus in undercook pork. NCC develops when humans accidentally ingest eggs. This occurs when feces of human carriers contaminates food, although the most important risk factor for the acquisition of cysticercosis is the proximity of a tapeworm carrier [4, 5]. Adult tapeworms shed proglottids, and each proglottid contains approximately 1000 to 2000 eggs. Once the hexacanth embryo reaches the parenchyma it forms cysticerci which undergo four stages of involution [6]. 

 The first is the vesicular stage characterized by a cyst with a translucent vesicular wall, transparent fluid, and a viable invaginated scolex. During this stage there is little host inflammatory reaction. The cyst then develops a thick vesicular wall, the fluid becomes turbid, and the scolex degenerates during the next stage, which is termed the colloidal stage. An intense inflammatory host response is seen and is reflected in the pathology which reveals varying degrees of acute and chronic inflammation [1, 6]. Radiographic examination reveals cystic lesions with edema and enhancement and seizures are common [7]. The cyst continues to degenerate as it moves into the granular stage which is characterized by a thick vesicular wall, degenerated scolex, gliosis, and little inflammatory host response. Ultimately the parasite transforms into coarse calcified nodules; the calcific stage [1, 2, 6].

## 2. Clinical Manifestations of Neurocysticercosis (NCC)

The clinical manifestations of NCC range from asymptomatic to life threatening. Within the CNS it can affect the parenchyma, subarachnoid space, or intraventricular system. Ocular and spinal disease occurs, but is less common. Therefore, the clinical manifestations are pleomorphic and dependant on the location, number, and stage of the cysts at presentation. NCC is the leading cause of adult-onset epilepsy in areas of the world where it is endemic, particularly in Latin America, Asia, and Africa [1]. Seizures are commonly generalized tonic-clonic or simple partial. Epilepsy occurs more frequent in patients with parenchymal disease, although it can occur in patients with cysts in the cortical sulci [1]. Seizures due to cysticercosis usually occur when the dying cyst incites an inflammatory reaction, but has been reported in the cystic stage. For many patients epilepsy may be the sole presentation of the disease with 50%–70% of patients experiencing recurrent seizures [8, 9]. 

 There are multiple ways that cysticercosis can cause seizures. As noted, seizures can occur early in the disease in the setting of intense inflammation associated with viable or degenerating cysts. They can also occur secondary to vasculitis and infarction which occurs in the setting of subarachnoid disease [10]. Lastly, increasing evidence implicates calcified NCC in the development and maintenance of seizures and epilepsy [11]. Patients who present with seizures in regions where infection with *T.* s*olium* is endemic commonly have calcified brain lesions observed on computerized axial tomography (CT) scan that are typical for NCC. In population-based studies calcified lesions on CT are much more common than viable cysts, and they are more prevalent in patients with epilepsy than they are in asymptomatic patients [11–13]. Strong evidence supports the role of calcified lesions in seizures; there is a high prevalence of cerebral calcifications in patients with seizures in the absence of other etiologies, and there is a positive correlation between endemic populations with increased proportions of calcification and seizure activity. In addition, individuals with calcified granulomas have increased risk of ongoing seizure [14–17]. 

**Figure 1:**
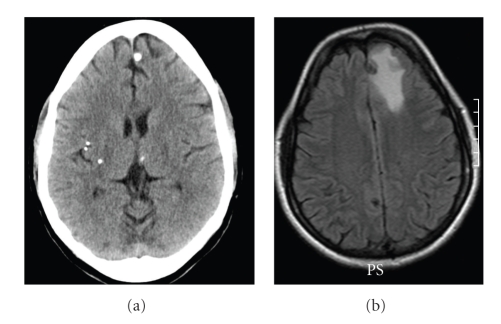
(a) Baseline CT scan demonstrating a dense calcification in the left frontal lobe as well as other calcifications. (b) MRI images that used fluid attenuated recovery after the patient had a seizure revealing perilesional edema.

 There has been increasing evidence that perilesional edema, which occurs episodically, is associated with seizures [14, 15, 18–20]. Perilesional edema appears as a bright signal using magnetic resonance imaging (MRI) FLAIR or T2 imaging (Figure 1). It is almost always accompanied by enhancement around the calcified focus [11]. Previously calcified NCC has been classified as the inactive form of the disease, suggesting that it is less important than other forms of NCC [21]. Recently a growing literature is finding that perilesional edema related to calcifications seems to be a relatively frequent phenomenon, with reports ranging from 23%–35% in literature [8, 14, 22]. The natural history or pathophysiology of perilesional edema is not yet known, but it appears that it recurs, and repeated episodes tend to be associated with the same lesions in a patient. In a recent prospective nested case-control study, 110 patients with seizures or headaches and calcified lesions in an endemic region were followed for recurrent seizures. Of those with recurrent seizures, perilesional edema was noted on MRI in 50% as opposed to 9% of asymptomatic matched controls [23]. This study suggests that perilesional edema is a common and potentially preventable cause of seizure in endemic regions. 

Although, seizures are the most common clinical manifestation of parenchymal NCC, focal neurologic signs have been reported and are usually related to the number, size, and location of the parasites in individuals with parenchymal disease. Intracranial hypertension can occur in patients with parenchymal NCC and is termed cysticercotic encephalitis [1, 24, 25]. This manifestation has been best described in children and young woman and is a result of the acute inflammatory response to massive cysticercal infection resulting in brain edema. Patients present with a syndrome characterized by clouding of consciousness, seizures, decreased visual acuity, headache, vomiting, and papilledema which can be subacute or acute in onset [1, 22]. These patients are treated with mannitol and corticosteroids in an attempt to control the inflammation and intracranial hypertension. Patients may even require decompressive temporal craniotomy. Those individuals with this form of NCC would not be candidates for antiparasitic agents, since treatment could exacerbate the inflammation and edema. Other causes of intracranial hypertension in patients with parenchymal NCC include the development of a large cyst that displaces midline structures or obstructs the flow of cerebrospinal fluid (CSF) in the cerebral aqueduct. 

 Psychiatric manifestations of NCC, such as depression and psychosis, have been described [26, 27]. A recent study found that patients admitted to a chronic inpatient psychiatric unit were more likely to have a positive serology for *T. solium* then healthy controls in the community. Of these inpatients, those with mental retardation were found to carry an increase risk of cysticercosis compared with patients with other psychiatric disorders. These patients were not carrying adult *Taenia spp*. in their stool and did not have CNS imaging, but the high prevalence of a positive cysticercosis serology in the inpatient psychiatric group suggests that there is a large proportion of cysticercosis in this group of patients [28]. Further studies are needed to explore the relationship between NCC and psychiatric disease. 

 Subarachnoid NCC is a common finding at autopsy, but when cysticerci find their way to the Sylvian fissure or the basilar cisterns the result can be devastating for the patient. The cysticercus larva (after embedding itself in the parenchyma) undergoes four stages of evolution: vesicular, vesicular colloid, granular nodular, and nodular calcified [1]. This evolution does not occur in the intraventricular or the subarachnoid form of NCC. The cisternal NCC is also-called the racemose type of cysticerosis. Racemose NCC refers to “aberrant proliferating cestode larvae” that manifest as solitary or multiple unencapsulated bladders that bud exogenously to form a multilocular cyst resembling a bunch of grapes [29]. The multiple cysts of the racemose type occur in nonconfining areas in and around the brain such as the suprasellar, sylvian and quadrigeminal cisterns. These cysts are nonviable, degenerated interconnected bladders of different sizes that often lack scolices, and can reach large sizes producing local mass effect. Arachnoiditis can occur with resulting communicating hydrocephalus secondary to either chronic inflammation or fibrosis of the arachnoid villi causing obstruction to the reabsorption of CSF or extension of the subarachnoid inflammatory reaction to the meninges at the base of the brain occluding the forminal of Luschka and Magendie [1, 30]. Cysticercotic arachnoiditis can lead to entrapment of cranial nerves in the inflammatory exudates that occur on the ventral aspect of the brain. Extraocular muscle paralysis, diploplia and papillary abnormalities are the result of entrapment of the ocular motor nerves. The optic nerves and the optic chiasm can also be encased within the exudates with subsequent development of decreased visual acuity and visual field defects [31, 32]. Acute aseptic meningitis associated with subarachnoid disease has been reported, but is rarely associated with fever and signs of meningeal irritation [1]. 

 Cerebrovascular complications of neurocysticercosis include cerebral infarction, transient ischemic attacks and brain hemorrhage [10, 33, 34]. The most common mechanisms by which NCC produces cerebrovascular disease are related to cerebral arteritis, mainly in those individuals with subarachnoid cysticercosis. Earlier clinical reports of cerebral infarction were secondary to small vessel involvement in NCC. In a recent study that examined 28 patients with subarachnoid disease 53% had angiographic evidence of cerebral arteritis with the middle cerebral and posterior cerebral arteries being the most commonly involved vessels associated with clinical stroke syndrome. The frequency of cerebral arteritis in subarachnoid cysticercosis seems to be higher than previously reported, and middle-size vessel involvement is a common finding [35]. 

 Clinical manifestations of ventricular NCC vary according to the size of the parasites, their location inside the ventricular system, and the coexistence of granular ependymitis [1, 36]. Lateral ventricles usually induce a syndrome of increased intracranial pressure which may be associated with focal neurological signs due to compression of adjacent structures [37, 38]. Patients with third ventricle cysticerci complain of progressively worsening headaches and vomiting due to developing obstruction or may present with sudden loss of conscious from acute hydrocephalus [39, 40]. Paroxysmal headache and vomiting secondary to intermittent obstruction at the level of the cerebral aqueduct has been described [1]. Cysts in the fourth ventricle can also cause subacute hydrocephalus that may be associated with signs of brainstem dysfunction secondary to compression of the fourth ventricle [41]. A well-described clinical presentation of fourth ventricle cyst is the Bruns' syndrome which is characterized by episodic headache, papilledema, neck stiffness, sudden positional vertigo induced by rotatory movements of the head, nausea and vomiting, drop attacks and loss of consciousness with rapid recovery and long asymptomatic periods [1]. Cysts in the third and fourth are a well-described cause of sudden death due acute obstructive hydrocephalus [41–43]. 

 A degenerating cyst in the ventricles can result in an inflammatory reaction throughout the ventricular system leading to granular ependymitis. When this occurs the cyst capsule can become fixed to the ventricular wall with strong adhesions and fibrosis [41]. Increased intracranial pressure due to hydrocephalus can occur if ependymitis occurs at the level of the cerebral aqueduct. These patients tend to have a more chronic course than those with cysts in the fourth ventricle [44]. 

 Spinal cord involvement in NCC is rare, accounting for 1%–5% of all cases [1, 45]. Spinal cord involvement can be intramedullary or extramedullary with the latter being more common. Intramedullary cysts are most common in the thoracic spine and patients usually present with gradual onset of myelopathy similar to the presentation of intramedullary tumors [46–52]. Extramedullary cysts or leptomeningeal NCC is usually an extension of subarachnoid disease which has migrated from the basilar cisterns. Cysts may be single or may form clumps of multiple cysts extending along the entire spinal canal [46, 47]. The resulting clinical picture is characterized by a combination of radicular pain and motor deficits of subacute onset and progressive course [1]. 

 Intraocular cysticerci may be located in the anterior chamber, the lens, the vitreous and the subretinal space, but the latter is the most common location. Cysts in the subretinal space can cause progressive decrease in visual acuity. Vitreous cysts can produce worsening vision with the perception of something moving within the eye. Cysts in the anterior chamber may induce a severe iridocyclitis, while retro-ocular intraorbital cysticerci may cause decreased visual acuity due to pressure on the optic nerve [1, 53, 54].

## 3. Radiological Manifestations

**Figure 2:**
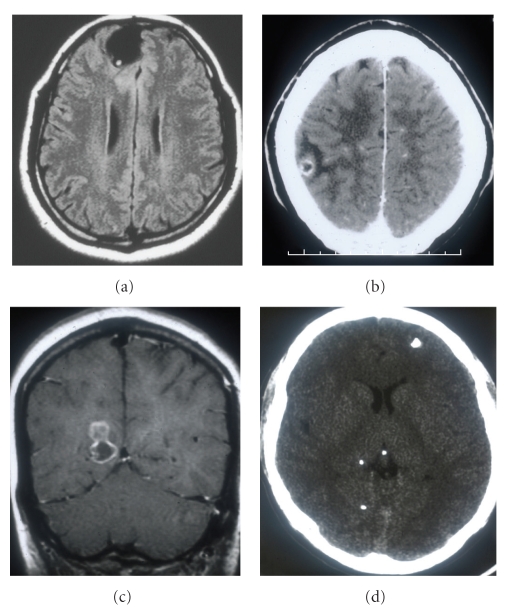
(a) MRI image revealing vesicular cysticerci (the central dot represents the scolex). (b) Cyst beginning to degenerate with perilesional edema and enhancement in the colloidal stage. (c) Cyst in the granular stage without perilesional edema. (d) CAT scan revealing multiple calcifications in the calcified stage.

Neuroimaging of parenchymal NCC depends on the stage of the development of the parasites. In the vesicular stage the cysticerci appear as cystic lesions within the brain parenchyma [7]. CT and MRI reveal that the cyst wall is thin and well demarcated from the parenchyma. The cysts lack perilesional edema and do not enhance after administration of contrast medium. There may be a bright nodule in their interior giving the lesion a “hole with dot” appearance that represents the scolex (Figure 2(a)) [55]. As the cysts begin to degenerate they appear as ill-defined lesions surrounded by edema which enhance after contrast medium administration. This is the colloidal stage of the cyst and represents the so-called “acute encephalitic phase” of NCC which likely represents an intense host reaction to the parasite (Figure 2(b)). MRI reveals a thick and hypointense wall with marked perilesional edema. The perilesional edema is best visualized on MRI with the fluid-attenuated inversion recovery (FLAIR) technique [7]. Granular cysticerci appear as nodular hyperdense lesions surrounded by edema or a rim of gliosis after contrast medium administration (Figure 2(c)) [55]. Calcified (dead) cysticerci appear on CT as small hyperdense nodules without perilesional edema (Figure 2(d)) or abnormal enhancement after contrast administration; these lesions are usually not visualized by MRI. Conversely, when calcified lesions are associated with perilesional edema and contrast enhancement, they are better seen by MRI [7, 55]. 

Cysticerci within the basilar cisterns are usually missed by CT scan and require MRI to adequately visualize them. While most subarachnoid cysts over the convexity of the cerebral hemispheres are small, lesions located in the Sylvian fissure may reach 50 mm or more in size; these parasites usually have a mulitlobulated appearance, displace neighboring structures, and behave as mass occupying lesions. Fibrous arachnoiditis commonly occurs in subarachnoid disease resulting in hydrocephalus which is the most common CT finding in subarachnoid NCC [7, 46]. Leptomeningeal enhancement at the base of the brain is observed best by MRI [56]. In general, the neuroimaging appearance of cerebrovascular complications is indistinguishable from cerebral infarcts from other causes [10]. 

 Ventricular cysts appear on CT images as cystic lesions. They are initially isodense with the CSF and are therefore not well visualized. However, their presence can be inferred from distortions of the ventricular system causing asymmetric or obstructive hydrocephalus [57]. In contrast, most ventricular cysts are well visualized by MRI because their signal properties differ from those of the CSF, particularly using FLAIR techniques [7]. They may also move within the ventricular cavities in response to movements of the patients' head (ventricular migration sign), a phenomenon that is best observed with MRI than with CT [58]. Occasionally, this finding facilitates the diagnosis of ventricular cysticercosis. 

 In patients with spinal NCC, CT may reveal symmetrical enlargement of the cord (intramedullary cysts) or pseudoreticular formations within the spinal canal (leptomeningal cysts). MRI reveals intramedullary cysticerci to be ring-enhancing lesions that may have an eccentric hyperintense nodule representing the scolex. Myelography still has a role in the diagnosis of patients with spinal leptomeningeal cysticercosis because it shows multiple filling defects in the column of contrast material corresponding to each cyst [59]. Leptomeningeal cysts may be mobile (changing their position according to the movements of the patient) [7, 59, 60].

## 4. Serology

Only tests based on detection of antibodies specific for *T. solium* antigens are reliable for clinical diagnosis and epidemiologic studies. To date, these are limited to those based on the use of purified glycoprotein antigens derived from *T. solium* cysticerci. The current assay of choice is the electroimmunotransfer blot (ETIB) using partially purified antigenic extracts [61, 62]. This assay has a specificity approaching 100% and a sensitivity of 94%–98% for patients with two or more cystic or enhancing lesions. A major limitation of these tests are frequent false negative results in patients with single intracranial cysticerci, in which fewer than 50% test positive. Sensitivity of specific antibody assays is also relatively low in patients with only calcified cysticerci [63]. 

 Detection of circulating parasite antigen reflects the presence of live parasites establishes the presence of ongoing viable infection and may permit quantitative verification of successful treatment [64–66]. Garcia and others have used Ag-ELISA based on the use of a monoclonal antibody (HP10) that reacts with a repetitive carbohydrate epitope found in excretory/secretory and surface antigens of living cysticerci [66, 67]. This assay had a sensitivity of 86% when tested on (CSF) samples from a series of 50 Peruvian patients with NCC [68]. The specificity of the assay is about 96% and it has been used to follow patients after treatment. Parasite antigen levels fell significantly by 3 months after treatment in patients with “cured” parenchymal disease after albendazole therapy [66]. This study found that the sensitivity is low in intraparenchymal NCC, especially in patients with only a few intraparenchymal cysts [66]. In a study examining patients with hydrocephalus and NCC the assay was positive in 14 of 29 patients, but negative in patients with calcifications [69]. A drop in antigen levels (serum and CSF) after treatment in subarachnoid disease has been reported in a small number of patients [70]. The management of subarachnoid disease is particularly complicated and the appropriate endpoint for treatment has not been established. Further studies employing this assay to follow patients with subarachnoid disease are needed. Recently a monoclonal antibody-based ELISA to detect *T. solium* antigens in urine has been described. The overall sensitivity of urine antigen detection for viable parasites was 92%, which decreased to 62.5% in patients with a single cyst. Most individuals with only calcified cysticercosis were urine antigen negative. This assay could be useful in diagnosis of NCC and evaluating the efficacy of treatment.

## 5. Treatment

### 5.1. Parenchymal Disease

Praziquantel and albendazole are antiparasitic agents that are effective against *T. solium* cysticerci killing between 60% and 85% of parenchymal brain cysticerci [71]. Most trials show greater cyst reduction with albendazole administration. However, most of these studies have been uncontrolled, observational imaging studies. The majority of studies evaluated praziquantel at a dosage of 50 mg/kg/d for 2 weeks, although studies describing a single day regimen have also been described [8, 72–83]. Higher doses have been used, but there is limited experience in literature [71, 72]. A dose of 15 mg/kg of albendazole for four weeks was initially employed, but later reduced to 15 days and then to one week [74, 76, 80, 81, 83–88]. Between the second and fifth day of treatment with an antiparasitic agent there may be an exacerbation of neurologic symptoms which has been attributed to inflammation secondary to killing of the cysticerci. Because of this inflammation steroids are generally administered in conjunction with albendazole or praziquantel to control the resulting edema [71]. It should be noted that steroids decrease the plasma level of praziquantel, but not albendazole [89]. 

 Randomized studies evaluating the clinical benefit of treatment have yielded conflicting data with some studies indicating a benefit and others failing to show a difference [90–94]. There has been much controversy whether cysticidal drugs modify the natural course of neurocysticercosis. In 2004 a randomized, placebo-controlled trial of treatment of adults with 20 or less viable parenchymal cysts and a history of seizures using albendazole demonstrated a reduction in seizures and enhanced resolution of cysts after treatment [95]. Although a landmark study, the treatment was not completely effective. The number of patients who became free of seizures was similar in the two groups, but the reduction in the number of the seizures among patients who received the treatment was significant in patients with generalized seizures, not in the group with partial seizures. Further studies are needed to determine whether longer or repeated courses of therapy will result in a decrease in seizures overall and leave patients with fewer remaining cysticerci. A recent meta-analysis confirmed that treatment of parenchymal NCC is clinically beneficial [96]. These authors concluded drug therapy results in better resolution of colloidal and vesicular cysticerci, lower risk for recurrence of seizures in patients with colloidal cysticerci, and a reduction in the rate of generalized seizures in patients with vesicular cysticerci. However, there were not sufficient data to determine conclusively the superiority of either albendazole or praziquantel as first-line treatment of NCC in this meta-analysis [96]. Despite the numerous studies, an optimal therapeutic regimen for neurocysticercosis has not been established. The evidence favors albendazole over praziquantel, but longer courses and repeated courses might be needed for patients with multiple cysts. Future trials should look to define the optimal therapeutic regimen. A recent prospective, randomized placebo, controlled trial examined combination therapy with albendazole and praziquantel versus albendazole alone in 110 children with seizsures and single enhancing lesions. There were no differences in recurrent seizures and resolution of the lesions. Larger studies are warranted with combination therapy in both parenchymal disease and extraparenchymal forms of neurocysticercosis [97].

 Single enhancing lesions have a good prognosis. Studies examining this group of patients have shown variable clinical results, probably due to the heterogeneity of morphology of single enhancing lesions. The most rigorous double-blinded randomized treatment trial showed an initial increase in seizure occurrence, but in a follow-up evaluation at two years there was a significant benefit of treatment [87, 88]. The previously mentioned meta-analysis found that enhancing lesions benefited from treatment with antiparasitics [96]. Solid nodular cysts that are degenerating have shown resolution with antiparasitic treatment. Calcified cysts need not be treated with antiparasitic agents [4, 71]. 

 Anticonvulsants are should be used to control seizures. Serum levels of phenytoin and carbamazepine may be lowered when given concomitantly with praziquantel [98]. 

 There is no proven effective treatment for perilesional edema associated with calcified lesions. Steroids can control symptoms, but there are no data that treatment with steroids will prevent recurrent edema [4, 14]. Methotrexate has been used in patients with recurrent perilesional edema to control the host inflammatory response as a steroid sparing agent in patients requiring long-term steroids [99, 100]. Patients with cysticercotic encephalitis should not be treated with cysticidal drugs because this may exacerbate the intracranial hypertension. Treatment should be aimed at relieving edema with corticosteroids (up to 32 mg per day of dexamethasone) and mannitol at doses of 2 mg/kg per day [2].

### 5.2. Extraparenchymal NCC

There are no controlled trials on the management of subarachnoid disease. In a series of patients treated with only CSF diversion, 50% died at a median follow-up of 8 years and 11 months [101]. Cysticidal drugs with steroids and shunting for hydrocephalus have been used with success in subarachnoid disease [46, 102, 103]. The host inflammatory reaction around the cysts may result in occlusion of leptomeningeal vessels resulting in stroke or hydrocephalus [9, 101]. Therefore, steroids must be used in conjunction with therapy [2, 4, 55]. Most experts consider subarachnoid NCC an indication for treatment with antiparasitic agents [71]. There is no consensus on the dose of antiparasitic agent or length of treatment for this form of NCC. A study of 33 patients with giant cysticerci in the Sylvian fissure treated with albendazole (15 mg/kg/d for 4 weeks) found only one single death from aplastic anemia at 59 months, with patients requiring several courses of therapy [102]. Therefore, a single course in patients with subarachnoid disease patients is probably inadequate and long-term therapy (months) might be required to treat some patients. Similarly, the optimal dose and duration of steroids has not been determined. Methotrexate has been used as a steroid sparing agent in subarachnoid disease in patients requiring long-term steroids and experiencing intolerable side effects [99]. 

 Therapy for ventricular disease needs to be individualized. Anthelmintic treatment of the fourth, third, and lateral ventricle has been reported [41, 104–107]. If hydrocephalus is present patients, should have a shunt placed prior to medical therapy [106]. Surgery has been the mainstay in this form of NCC [39, 108]. There is a growing literature supporting flexible neuroendoscopy to remove approachable subarachnoid cysts and cysts lodged in the lateral, third, and fourth ventricles [109–112]. Cysts that enhance on MRI may not be suitable for endoscopic removal. 

 It is important to recognize that the management of NCC is complicated and involves antiinflammatory agents, antiparasitic drugs, and in some cases surgery. It should be managed by physicians knowledgeable in this field.
